# Disturbed Balance of Inhibitory Signaling Links Hearing Loss and Cognition

**DOI:** 10.3389/fncir.2021.785603

**Published:** 2022-01-06

**Authors:** Marlies Knipper, Wibke Singer, Kerstin Schwabe, Gisela E. Hagberg, Yiwen Li Hegner, Lukas Rüttiger, Christoph Braun, Rüdiger Land

**Affiliations:** ^1^Department of Otolaryngology, Head and Neck Surgery, Tübingen Hearing Research Center (THRC), Molecular Physiology of Hearing, University of Tübingen, Tübingen, Germany; ^2^Experimental Neurosurgery, Department of Neurosurgery, Hannover Medical School, Hanover, Germany; ^3^Department of Biomedical Magnetic Resonance, University Hospital Tübingen (UKT), Tübingen, Germany; ^4^High-Field Magnetic Resonance, Max Planck Institute for Biological Cybernetics, Tübingen, Germany; ^5^MEG Center, University of Tübingen, Tübingen, Germany; ^6^Center of Neurology, Hertie-Institute for Clinical Brain Research, University of Tübingen, Tübingen, Germany; ^7^Department of Experimental Otology, Institute for Audioneurotechnology, Hannover Medical School, Hanover, Germany

**Keywords:** inhibitory strength, fast auditory processing, PV interneurons, dementia, tinnitus, deafness, BDNF, hearing loss

## Abstract

Neuronal hyperexcitability in the central auditory pathway linked to reduced inhibitory activity is associated with numerous forms of hearing loss, including noise damage, age-dependent hearing loss, and deafness, as well as tinnitus or auditory processing deficits in autism spectrum disorder (ASD). In most cases, the reduced central inhibitory activity and the accompanying hyperexcitability are interpreted as an active compensatory response to the absence of synaptic activity, linked to increased central neural gain control (increased output activity relative to reduced input). We here suggest that hyperexcitability also could be related to an immaturity or impairment of tonic inhibitory strength that typically develops in an activity-dependent process in the ascending auditory pathway with auditory experience. In these cases, high-SR auditory nerve fibers, which are critical for the shortest latencies and lowest sound thresholds, may have either not matured (possibly in congenital deafness or autism) or are dysfunctional (possibly after sudden, stressful auditory trauma or age-dependent hearing loss linked with cognitive decline). Fast auditory processing deficits can occur despite maintained basal hearing. In that case, tonic inhibitory strength is reduced in ascending auditory nuclei, and fast inhibitory parvalbumin positive interneuron (PV-IN) dendrites are diminished in auditory and frontal brain regions. This leads to deficits in central neural gain control linked to hippocampal LTP/LTD deficiencies, cognitive deficits, and unbalanced extra-hypothalamic stress control. Under these conditions, a diminished inhibitory strength may weaken local neuronal coupling to homeostatic vascular responses required for the metabolic support of auditory adjustment processes. We emphasize the need to distinguish these two states of excitatory/inhibitory imbalance in hearing disorders: (i) Under conditions of preserved fast auditory processing and sustained tonic inhibitory strength, an excitatory/inhibitory imbalance following auditory deprivation can maintain precise hearing through a memory linked, transient disinhibition that leads to enhanced spiking fidelity (central neural gain⇑) (ii) Under conditions of critically diminished fast auditory processing and reduced tonic inhibitory strength, hyperexcitability can be part of an increased synchronization over a broader frequency range, linked to reduced spiking reliability (central neural gain⇓). This latter stage mutually reinforces diminished metabolic support for auditory adjustment processes, increasing the risks for canonical dementia syndromes.

## Introduction

Hearing loss is a very common problem in the aging population of industrial societies. Globally, an estimated 1.57 billion people had hearing loss in 2019, accounting for one in five people (20.3%) (Goman and Lin, 2016; Collaborators, 2021). The problem is even worse among the elderly; more than 25% of people over 60 suffer from hearing loss. Hearing loss not only impairs communication, social interaction, and quality of life, but has also been identified as a common risk factor for cognitive decline and Alzheimer’s disease (Lin F. R. et al., 2011; Livingston et al., 2017; Montero-Odasso et al., 2020). However, at the moment there has been no confirmation of a direct link between hearing loss and cognitive decline, which is, instead, currently assumed to be based on differences in myelination (Lin H. W. et al., 2011), auditory cognitive dysfunctions, or neurodegenerative processes (Fortunato et al., 2016; Uchida et al., 2019; Johnson et al., 2021).

Here, we review how impaired auditory input can affect the excitatory/inhibitory balance within the central auditory system and suggest that hearing loss and cognitive decline may be linked through changes in the excitatory/inhibitory balance associated with the functional attenuation of distinct auditory fiber types. We further suggest that these changes in the excitatory/inhibitory balance may, in turn, influence neurovascular coupling, possibly further affecting cognitive function in aging.

In the following, we lay out this idea in more detail. First, we provide an overview of the development of inhibitory GABAergic circuits (see Section “Maturation of GABA-Responsive Neurons Prior to Hearing Onset”). Second, we describe the role that different auditory nerve-fiber types might play during development and in regulating the excitatory/inhibitory balance in the auditory system (see Section “Activity-Dependent Maturation of GABAergic Inhibitory Circuits After Hearing Onset: The Potential Role of Auditory Nerve Fibers”). We then discuss the role of fast auditory processing (see Box 1) may play for maintaining the excitatory/inhibitory balance and sustaining or improving stimulus resolution and discrimination above noise after, e.g., mild acoustic trauma or hearing deficits. We hypothesize that fast auditory processing is a prerequisite for an increased central neural gain process (see Box 2). Within this multi-level reinforcing framework, activity dependent brain-derived neurotrophic factor (BDNF) and fast spiking PV-IN contribute to improving central auditory plasticity (see Section “Altered Excitation and Inhibition After Acoustic Trauma and Age-Related Hearing Loss Are Linked to Increased Central Neural Gain”). In other auditory impairments such as acute acoustic trauma, deafness, or tinnitus, hyperexcitability may be the result of reduced (tonic) inhibitory strength (see Box 3) following less-developed or impaired fast auditory processing and subsequent failure to recruit BDNF and PV-IN dependent increased central neural gain (see Section “Altered Excitation and Inhibition in Acute Acoustic Trauma, Deafness, and Tinnitus: Lost Fast Auditory Processing”). Further, we discuss how a decline in fast auditory nerve processing, when critically reducing tonic inhibitory strength in auditory nuclei, might be linked to cognitive deficits or autism (see Section “Altered Excitation and Inhibition Following Diminished Fast Auditory Processing Linked to ‘Central’ Hearing Loss”). Finally, we point to a possible role for inhibitory circuits in regulating neurovascular hemodynamic responses as a stress-sensitive process. Ultimately, under these conditions, deficits in central processing and auditory cognitive brain dysfunctions are expected. Sustained fast auditory processing and tonic inhibitory strength may be a key signature that bridge hearing and cognition (see Section “Coupling of Inhibitory/Excitatory Circuit Activation to Cerebral Blood Flow”).

**Box 1. Fast auditory processing.**

We define fast auditory processing as the increase in auditory acuity that is linked to lowering of hearing thresholds, increased suprathreshold ABR waves I and IV, the shortening of first spike latencies, and widening of response dynamic range with auditory experience, all shown in DCN neurons (Eckert et al., 2021), IC neurons (Chumak et al., 2016), and auditory cortex neurons (de Villers-Sidani et al., 2007; Xu et al., 2010). Because high-SR auditory fibers determine the threshold of the auditory-nerve response measured by the compound action potential (CAP) (Bourien et al., 2014), and these highly active fibers enable the shortest-latency auditory responses whatever the characteristic frequency (Meddis, 2006; Heil et al., 2008), we hypothesize that fast (high-SR) auditory fibers are also responsible for lowering of thresholds and shortening of latency of cortical auditory responses with auditory experience (de Villers-Sidani et al., 2007). This improved auditory acuity occurs after hearing onset in rodents ∼P11 (de Villers-Sidani et al., 2007) and in humans likely between the 27th embryonic week and 6th to 12th months after birth (Neville and Bavelier, 2002). Moreover, fast auditory processing is a likely prerequisite for precise temporal auditory coding, pure tone pitch perception, and frequency discrimination – all characteristics that are required for proper speech intelligibility (Oxenham, 2018) and experience-driven auditory attention (Addleman and Jiang, 2019).

**Box 2. Increased central neural gain.**

We define increased central neural gain as the identifiable network homeostasis that increases stimulus-evoked synchronous neural activity at the level of the inferior colliculus (IC) (ABR wave IV) relative to its input at the level of the auditory nerve (ABR wave I). Increased central neural gain can occur following, e.g., auditory deprivation (age, injury, and trauma) or sound enrichment. As a multi-level framework, central neural gain includes a positive auditory feedforward and positive fronto-striatal feedback cycle that require co-activation. Mechanistically increased central neural gain likely requires a reinforcement process, as it is also known from auditory perception or improved task performance [for a review see Irvine (2018a)]. During improved task performance, for example, PV-IN activity in frontal brain regions contributes to feedforward inhibition that narrows the window for temporal summation of EPSPs and action potential initiation in, e.g., principle neurons (Pouille and Scanziani, 2001). Through feedback inhibition, a sharpening of receptive fields and pattern separation is initiated (Leutgeb et al., 2007). During this process, stimulus resolution and discrimination above noise, as well as neuronal output activity, is facilitated in sensory systems through, e.g., cortical or prefrontal brain inhibitory neurons that specifically suppress the firing of other inhibitory neurons (Caraiscos et al., 2004; Cardin et al., 2009; Pi et al., 2013; Hu et al., 2014; Kim et al., 2016; Chen et al., 2017). This results in enhanced stimulus response reliability, decreased response variability, and increased signal-to-noise ratio (Sohal et al., 2009; Zhu et al., 2015; Espinoza et al., 2018).

**Box 3. Tonic inhibitory strength.**

We define tonic inhibitory strength as a sustained form of microcircuit network suppression. In the case of loss of inhibitory strength, spontaneous firing rate would increase without increasing a stimulus-evoked spike output. In the cerebellar cortex, such a phenomenon was described after a blockade of tonic inhibition in granule cells (Duguid et al., 2012). It is currently assumed that tonic inhibition suppresses spontaneous activity through a reduction of the neuronal input resistance and membrane time constants, thereby improving stimulus discrimination above noise (Caraiscos et al., 2004). The ability of tonic inhibition to change conductance in many neurons is assumed to require perisynaptic and extrasynaptic δ subunit-containing GABA_*A*_ receptors, which are likely activated through fast-spiking, parvalbumin (PV)-expressing and soma-inhibiting interneurons (IN) (Ferando and Mody, 2015). When tonic PV-IN activity is functionally impaired, the rapid increase in bursting reduces the signal-to-noise ratio (Duguid et al., 2012). The pathological hyper-synchronization resembles electrical seizure activity (Rossignol et al., 2013; Fröhlich, 2016; Hsieh et al., 2017), and possibly enhances baseline spontaneous gamma power, reduces evoked gamma power (Mamashli et al., 2017), and can in this way also disturb the signal-to-noise ratio.

This article should not be understood to be all-encompassing, but a reference to the respective research interests of the authors, in order to increase awareness that the brain’s hyperexcitability can have different origins, dependent on whether the inhibitory strength generated in microcircuits with auditory experience is maintained or not. We finally deliberately propose this view as a “general concept.” In the best case, we hope to inspire an interdisciplinary effort to examine the suggested hypothesis in the context of various auditory diseases. Only then can personalized intervention strategies be successfully implemented to overcome such devastating disorders as dementia, to which auditory cognitive deficits may contribute.

## Maturation of Balanced Inhibitory/Excitatory Circuits in the Auditory System

### Maturation of GABA-Responsive Neurons Prior to Hearing Onset

A balance between excitation and inhibition is crucial for the precise encoding of complex sounds. In this context, it is important to consider that balanced excitatory/inhibitory neuronal activity develops only after hearing onset. Early in neonatal development, radially migrating neurons that originate in the ventricular zone of the pallium (cortex) give rise to glutamatergic pyramidal neurons, while a second population of tangentially migrating neurons, originating in the ventricular zone of the subpallium (subcortical telencephalon), give rise to GABA-producing local-circuit neurons (Marin and Rubenstein, 2001). Tangentially migrating GABAergic neurons, which target either higher-level cortical regions or lower-level brain regions posterior to the midbrain, originate from different brain regions and are characterized by different paired-box (Pax) homeobox genes. The GABAergic interneurons that migrate from the subpallium to cortical regions are thought to express Pax6 (Maricich and Herrup, 1999; Marin and Rubenstein, 2001), while the GABAergic interneurons that migrate from ventricular zones to lower brain levels posterior to midbrain regions express Pax2 (Nornes et al., 1990; Maricich and Herrup, 1999; Rowitch et al., 1999; Fotaki et al., 2008).

**FIGURE 1:**
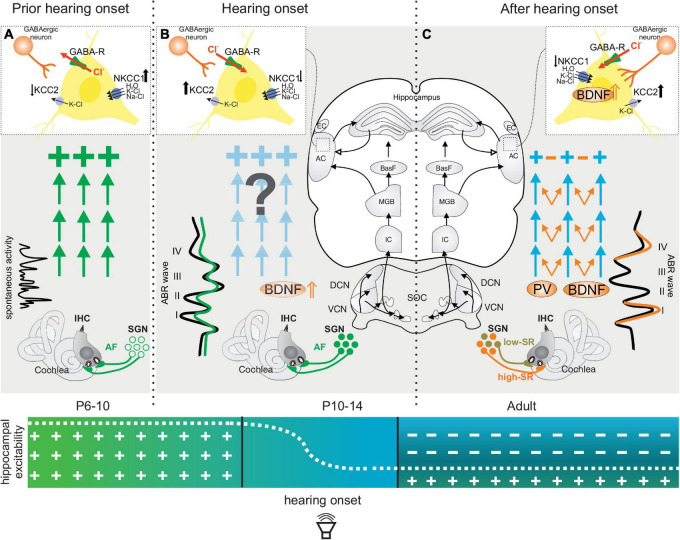
Maturation of neuronal inhibitory circuits in the auditory system prior to **(A)**, during **(B)**, and after **(C)** hearing onset. **(A)** Prior to hearing onset, when GABAergic neurons (inset, light red cell) do not yet contact target cells, GABA-responsive pyramidal neurons favor a chloride efflux (inset, red arrow) and thereby a depolarization of GABA-responsive neurons. A high intracellular chloride concentration in these cells is supported by low levels of neuronal potassium chloride co-transporter type 2 (↓ KCC2) and elevated sodium-potassium-chloride co-transporter type 1 (NKCC1↑). At this time, an initial hyper-excitability dominates (large green crosses) and IHCs show only spontaneous firing. **(B)** Shortly before hearing onset, BDNF is upregulated in the cochlea (Wiechers et al., 1999) and a switch in the effect of GABA from depolarizing to hyperpolarizing occurs (Lohrke et al., 2005) (**B** inset, GABAergic neuron and red inward arrow), accompanied by a reduced NKCC1↓ and an increased KCC2↑ expression (**B**, inset). This may already be driven by auditory input (Shibata et al., 2004) (**B**, green ABR wave on the left). **(C)** A switch of the GABA action from excitatory/depolarizing to inhibitory/hyperpolarizing is initiated in projection neurons after hearing onset, (P10–14). This time period parallels the maturation of the high-SR (**C**, orange fiber) and low-SR auditory nerve fibers (**C**, green fibers). The switch of GABA from excitatory to inhibitory (**B,C**, inset) is initiated by an upregulation of KCC2 ↑ after hearing onset, (P10–14). KCC2 promotes a lower concentration of intracellular chloride in GABA-responsive neurons and consequently promotes hyperpolarizing inhibitory postsynaptic potentials upon GABA stimulation. As up-regulation of KCC2 is driven by BDNF, and BDNF is shown to foster the maturation of parvalbumin networks (**C**, orange arrows), fast (high-SR) auditory fiber processing may trigger the stimulus-evoked release of BDNF from auditory projection neurons and subsequently drive synaptogenesis of complex parvalbumin-expressing GABAergic interneuron networks toward sharply clustered brain circuits that respond precisely to auditory stimuli (**C**, orange arrows, orange ABR wave). ABR, auditory brainstem response; IHC, inner hair cell; SGN, spiral ganglion neuron; VCN, ventral cochlear nucleus; DCN, dorsal cochlear nucleus; SOC, superior olivary complex; IC, inferior colliculus; MGB, medial geniculate body; BasF, basal Forebrain; AC, auditory cortex; EC, entorhinal cortex; PV, parvalbumin.

In rodents, the radially migrating excitatory neurons, followed by the tangentially migrating GABAergic neurons, reach their final destinations around birth (Marin and Rubenstein, 2001; Markram et al., 2004; Li et al., 2018). At this stage, GABA at the GABA-responsive neurons still acts in excitatory fashion (Figure 1A), corresponding with a transient, initial hyperexcitability phase (Figure 1A, green arrows and crosses). In the cortex, this occurs after migration of GABAergic neurons to the cortical plate (Marin and Rubenstein, 2001).

**FIGURE 2:**
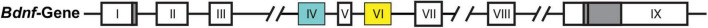
Schematic drawing of the rodent *Bdnf* gene, which is composed of eight non-coding exons (I–VIII) that are individually transcribed and alternatively spliced to the protein-encoding exon IX. *Bdnf* exon IV and VI are directly or indirectly regulated by changes in neuronal activity. In BDNF-Live-Exon-Visualization (BLEV) mice (Matt et al., 2018; Singer et al., 2018b), BDNF exon IV and VI are individually labeled with either cyan (exon IV) or yellow (exon VI) fluorescence protein in regions of activity-dependent translation of BDNF. Modified after (Aid et al., 2007; Singer et al., 2018b).

The initial hyperexcitability is due to the high intracellular chloride concentration of GABA-responsive neurons, which when activated by GABA favors a chloride efflux and thereby a depolarization of the neuron (Ben-Ari et al., 1989; Marin and Rubenstein, 2001; Ben-Ari, 2002) (Figure 1A, inset red arrow Cl^–^). In the auditory pathway of rodents, it has been shown that, early in postnatal development and prior to hearing onset, a high intracellular chloride concentration ([Cl^–^]_*i*_) is maintained in most neurons, ensured by the sodium-potassium-chloride co-transporter type 1 (NKCC1) (Figure 1A, inset NKCC1⇑). Hence, Cl^–^-mediated synaptic activities cause a depolarizing response (Balakrishnan et al., 2003; Cherubini et al., 2011; Friauf et al., 2011). Briefly, prior to hearing onset, around P5-P6 in rodents (Lohrke et al., 2005), or possibly driven by auditory experience, as shown after unilateral or bilateral cochlear ablation (Shibata et al., 2004), a switch of GABA-responsive neurons occurs and the effect of GABA changes from depolarizing to hyperpolarizing (Figure 1B). The switch from depolarizing to hyperpolarizing responses of GABA-responsive neurons is linked to an enhanced expression of the neuronal potassium chloride co-transporter type 2 (KCC2), which leads to a low concentration of intracellular chloride and, consequently, to a hyperpolarizing inhibitory postsynaptic potential upon GABA stimulation (Kandler and Gillespie, 2005) (Figure 1B, inset GABA KCC2⇑). Accordingly, the levels of the KCC2 transporter in the brainstem and ascending associated hippocampal regions are expectedly low before hearing onset (Figure 1A, inset KCC2⇓), and increase from the first postnatal week onward in a region-specific pattern (Figure 1B, inset KCC2⇑), as shown for the ascending auditory pathway (Lohrke et al., 2005) and other brain regions (Kandler and Friauf, 1995; Rivera et al., 1999; Friauf et al., 2011; Hirtz et al., 2011; Watanabe and Fukuda, 2015). This is the time when an upregulation of activity-dependent *Bdnf* transcripts is observed in cochlear spiral ganglion neurons (SGN) and at lower auditory brain levels (Singer et al., 2014) (Figure 1B, BDNF⇑). BDNF is suggested to modulate GABAergic synapses by postsynaptic regulation of chloride transport (Wardle and Poo, 2003). Since BDNF drives the upregulation of KCC2 expression (Ben-Ari et al., 2012) and both BDNF (Aid et al., 2007) and KCC2 (Fiumelli et al., 2005; Wake et al., 2007) are controlled by neuronal activity (Awad et al., 2018), the switch from depolarizing to hyperpolarizing responses of projecting neurons may start in the ascending auditory pathway and associated limbic frontal brain regions in response to an upregulation of activity–driven *Bdnf* transcripts. Activity-driven *Bdnf* transcripts are the result of independently transcribed non-coding exon IV and exon VI that, from a total of eight non-coding exons (I–VIII), are spliced to a common protein-encoding exon (IX) (Timmusk et al., 1993; Aid et al., 2007; Vaghi et al., 2014) (Figure 2). Both exon IV (Figure 2, cyan) and exon VI (Figure 2, yellow) comprise promoters directly or indirectly regulated by neuronal activity (Hong et al., 2008; Dieni et al., 2012; West et al., 2014; Chacon-Fernandez et al., 2016; Tuvikene et al., 2016).

In analogy to the visual system, the upregulation of BDNF in the cochlea and ascending pathway prior to hearing onset is suggested to occur in response to the influences of top-down hypothalamic corticotropin-releasing factor (CRF) (Knipper et al., 2015; Vetter, 2015). In response to these changes, spontaneous glutamate release from inner hair cells (IHCs), long predicted to play a crucial role in the maturation of central auditory circuits (Friauf and Lohmann, 1999; Kandler and Gillespie, 2005; Kandler et al., 2009; Hirtz et al., 2011), could activate *Bdnf* promoters in SGN to drive the depolarizing-to-hyperpolarizing switch in a bottom-up direction within the ascending auditory circuits both prior to and following hearing onset (Figure 1B, BDNF⇑). This would prepare auditory microcircuits for the subsequently occurring experience-driven synaptogenesis of perisomatic GABAergic contacts with the ascending microcircuits (next section). Taking this into account, differences in the vulnerability of cochlear neurons related to altered cochlear BDNF (Meltser et al., 2014) or CRF levels (Graham and Vetter, 2011) may be reconsidered in future studies in the context of changes in cochlear BDNF or CRF might potentially affect GABAergic inhibitory strength in the auditory pathway.

In summary, prior to the first auditory experience and during hearing onset, an initial period of hyperexcitability exists, with excitatory activity dominating over inhibitory activity. Within this transient time period, GABA-responsive neurons have reached their target regions but still react with depolarizing responses, due to the low level of neuronal KCC2 and high [Cl^–^]_*I*_ concentrations.

### Activity-Dependent Maturation of GABAergic Inhibitory Circuits After Hearing Onset: The Potential Role of Auditory Nerve Fibers

When considering possible events that may be causally linked to inhibitory GABAergic circuit formation in the auditory system, it is interesting to focus on the differential maturation times of different types of auditory nerve fibers. These roughly 30,000 auditory nerve fibers in the mammalian inner ear receive signals from individual IHC *via* ribbon synapses (Spoendlin, 1969; Liberman, 1980; Nadol, 1988), and transmit the signals further to the subsequent structures of the central auditory pathway. Auditory nerve fibers differ in their spontaneous firing rates (SR) and sound level thresholds and can be divided into at least two types. The low-SR, high-threshold auditory fibers, characterized by a low spontaneous firing rate of <18 spikes/s, comprise around 40% of all auditory nerve fibers, and the high-SR low threshold fibers, which have a high spontaneous firing rate >18 spikes/s, comprise the remaining 60% (Sachs and Abbas, 1974; Liberman, 1982; Yates, 1991; Merchan-Perez and Liberman, 1996; Glowatzki and Fuchs, 2002; Grant et al., 2010). SGNs with different SRs form synapses at different modiolar-to-pillar positions along the basolateral surface of IHCs (Liberman, 1982).

The mechanism that leads to maturation and differentiation of the distinct SR characteristics of auditory nerve fiber types is still under debate. A recent study of Shrestha et al. (2018), identified characteristic patterns of genes in SGNs of mature mice (P25), that from their anatomical position across the IHCs were characteristic for SGN fates of high, middle, and low-SR auditory nerve fibers. They showed that prior to hearing onset, representative genes for the SGN fate of low-SR auditory nerve fibers are shaped out of pre-existing SGNs that have the SGN fate typical of high-SR auditory nerve fibers. This happens over time - between P3 and P8 - in an activity-dependent manner (Shrestha et al., 2018). This would mean that prior to hearing onset, the SGN fate of high-SR would precede that of low-SR fibers. In contrast, when auditory nerve activity was recorded at the time of hearing onset —in mice around P11—, their multivesicular excitatory postsynaptic currents (EPSCs) with lower amplitudes preceded and contrasted with monophasic EPSCs with sharp rise times and 10 times larger amplitudes that were recorded after hearing onset at P19–P21 (Grant et al., 2010). It was speculated that low EPSC amplitude distributions may represent fibers with low spontaneous rates (Figure 1C, light green fiber), ‘as most synaptic events may be insufficiently large to activate APs.’ In contrast, fibers with monophasic EPSCs and larger amplitudes may correspond to high-SR ANF (Figure 1C, orange fiber), as most excitatory postsynaptic potentials (EPSPs) may activate APs (Glowatzki and Fuchs, 2002; Grant et al., 2010). This suggested a substantial shift in the mode of transmitter release in IHCs, from preferential release of single vesicles in IHCs in immature animals during hearing onset, to preferential and coordinated release of seven to nine vesicles in IHCs from hearing animals (Grant et al., 2010).

In addition, medial efferents that form transient cholinergic synapses with IHCs during the first postnatal week (Glowatzki and Fuchs, 2000) may contribute to the different SR of auditory fibers or SGN fate (Knipper et al., 2015, review), as they alter the precision of spike timing of auditory fibers (Johnson et al., 2011, 2013). In analogy to the visual system, an altered spike timing precision may initiate, e.g., a hypothalamic top-down feedback signal to cochlear neurons, resulting in BDNF upregulation, here suggested to potentially influence the inhibitory strength of ANF (see Section “Maturation of GABA-Responsive Neurons Prior to Hearing Onset”). This may be analogous to the BDNF- and dopamine-induced improvement of retinal acuity through receptive-field re-organization of retinal ganglion cells (RGCs) (Sinclair et al., 2004; Witkovsky, 2004; Landi et al., 2009). Moreover, lateral dopaminergic feedback to auditory nerve fibers may influence high-SR rate characteristics, as shown by auditory nerve recording under dopaminergic receptor blockade (Ruel et al., 2006), as previously discussed in detail (Knipper et al., 2015). Here, a dopamine-induced modification of GABA_*A*_ receptor-mediated tonic inhibition may be considered (Crunelli and Di Giovanni, 2014).

Overall, it can be concluded that several events may contribute to the different physiological functions and firing-rate characteristics of auditory nerve fibers in the mature auditory system (i) IHC-driven synaptic events that mature during hearing onset, (ii) differences in cochlear IHC output activity through differential maturation of efferent feedback to auditory fibers, as well as (iii) differences in the genetic fate of SGNs. After hearing onset, fast auditory processing (Box 1) matures with high-SR auditory nerve fiber responses that determine the threshold of compound action potentials of the auditory nerve (Bourien et al., 2014) and are responsible for the shortest latencies seen in auditory responses at any given characteristic frequency, suggesting that they determine the perceptual thresholds (Meddis, 2006; Heil et al., 2008). The process of high-SR auditory nerve fiber maturation is thus likely related to the increased ABR wave amplitudes and their shortened latencies after hearing onset (∼P11) in rodents (e.g., Song et al., 2006) as well as to the sharpening of cortical receptive fields observed in rodents between the 2nd and 3rd postnatal week (Lendvai et al., 2000; de Villers-Sidani et al., 2007; Takesian et al., 2018).

The sharpening of cortical receptive fields, i.e., narrower bandwidth responses, occurs for all sensory cortices, including the auditory cortex (Xu et al., 2010) as a result of the stimulus-evoked release of BDNF from cortical pyramidal neurons (Itami et al., 2007; Hong et al., 2008; Xu et al., 2010; Lehmann et al., 2012; Griffen and Maffei, 2014; Kimura and Itami, 2019) (Figures 1B,C, BDNF ⇑). The released BDNF appears to drive the synaptogenesis of a complex network from peri-somatic and dendritic fast-spiking PV-INs that contact cortical pyramidal neurons (Hong et al., 2008; Xu et al., 2010; Lehmann et al., 2012) (Figure 1C, blue, orange arrows and inset). In accordance with this, between the 2nd to 3rd postnatal week, PV-IN staining levels increase in ascending auditory circuits and their cortical projections (Lohmann and Friauf, 1996), and inhibitory strength increases in microcircuits, as also observed in other sensory systems (Lohmann and Friauf, 1996; Itami et al., 2007; Xu et al., 2010; Lehmann et al., 2012; Kimura and Itami, 2019) (Figure 1C, inset perisomatic GABAergic contacts increase).

Important to mention here is that the overall process of maturation of fast auditory processing appears to be dispensable for basal hearing function. Thus, when BDNF was deleted in GABAergic precursor neurons in the brainstem of mice under the Pax2 promoter, and despite normal hearing thresholds based on measuring outer hair cell function, supra-threshold auditory nerve (ABR wave I) amplitudes remained low and the late ABR wave IV was delayed, indicating that fast auditory processing may have not matured properly (Zuccotti et al., 2012). As a result, profound deficits in precise auditory acuity occurred (Zuccotti et al., 2012; Chumak et al., 2016; Eckert et al., 2021), and was evident in the reduced dynamic range, elevated spontaneous firing rates (SFR), delayed first-spike latency, and reduced inhibitory strength in the dorsal cochlear nucleus and inferior colliculus (IC) (Chumak et al., 2016; Eckert et al., 2021). Under these conditions also, dendritic filopodia extensions of PV-IN positive interneurons were few in the auditory cortex and hippocampus in comparison to wild-type animals, despite PV-IN being normal in numbers (Eckert et al., 2021). This suggested that in rodents during the first postnatal weeks, the maturation of fast auditory processing (Figure 1C, high-SR in orange), the maturation of inhibitory strength in the ascending auditory pathway (Zuccotti et al., 2012; Chumak et al., 2016; Eckert et al., 2021) (Figure 1C, PV, orange arrows), and the stimulus-evoked release of BDNF from cortical pyramidal neurons (Figure 1C, inset, BDNF⇑) that drives the synaptogenesis of fast-spiking PV-IN microcircuits (Xu et al., 2010) are events that depend on experiencing sound.

To obtain an idea when this critical time period of maturation of inhibitory strength occurs in auditory and associated circuits in humans, we have to consider that the fast inhibitory PV-IN activity regulates not only higher cortical microcircuit functions (Griffen and Maffei, 2014; Kimura and Itami, 2019), but also feedforward and feedback inhibition (Hu et al., 2014, 2018) and its functional correlates, i.e., the gamma- and beta frequency oscillations (Cardin et al., 2009; Sohal et al., 2009; Gill and Grace, 2014; Chen et al., 2017). In children, increased gamma oscillations, associated with feedforward inhibition, occur at the age of less than 6 months, and are followed by increased beta oscillations, reflecting feedback inhibition (Sowell et al., 2001; Ortiz-Mantilla et al., 2016). At the same time, the latencies of the sound-induced auditory brainstem response (ABR) become shorter (Neville and Bavelier, 2002; Sharma et al., 2016). In parallel, functional brain connectivity increases from the 6th month of age onwards, when the neural activity becomes more clustered and specific for sensory modalities (Sowell et al., 2001; Neville and Bavelier, 2002; Ortiz-Mantilla et al., 2016) (Figure 1B, blue arrow). The clustering of sensory modalities, in turn, is accompanied by an enhanced comprehension of speech in noise (Obleser et al., 2007; Youssofzadeh et al., 2018), all progressing with a gradually improved capacity for auditory discrimination and temporal discrimination (Sowell et al., 2001; Fox et al., 2012; Miller and Buschman, 2013; Ankmnal Veeranna et al., 2019).

We thus conclude that auditory experience-dependent maturation processes of high-SR auditory nerve fibers in the auditory system are critical for the maturation of fast auditory processing, including the formation of activity-driven, fast inhibitory PV-IN microcircuits. Only then is the neuronal network implemented for a fine-grained resolution of sound discrimination, temporally precise hearing, and fast discrimination of novel auditory information (Figures 1B,C).

## Altered Excitation and Inhibition After Acoustic Trauma and Age-Related Hearing Loss Are Linked to Increased Central Neural Gain

**FIGURE 3:**
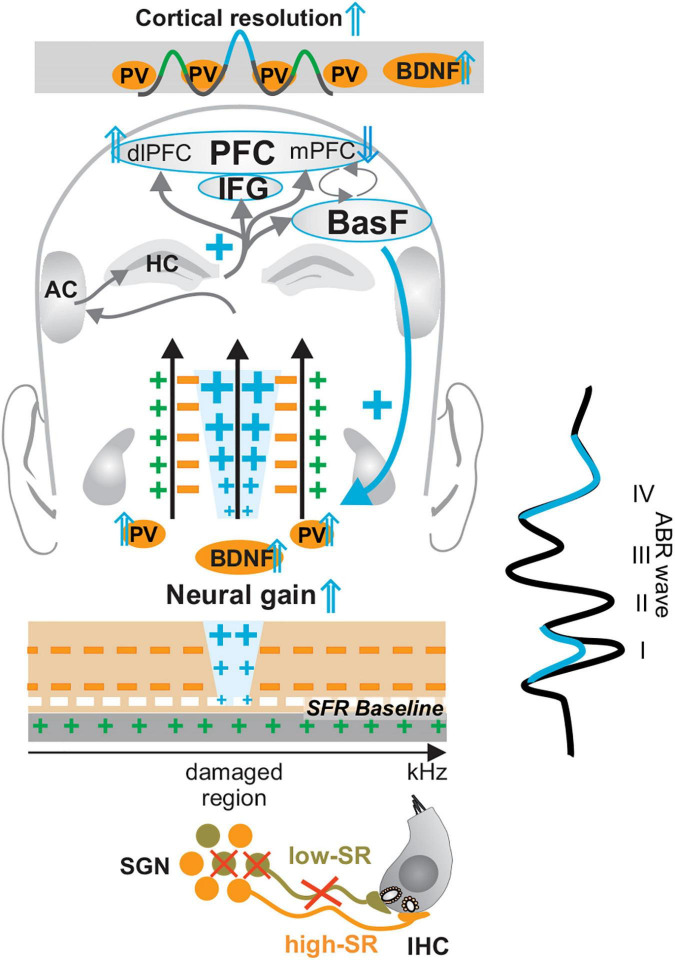
Central neural gain mechanism following mild acoustic trauma and aging. When the numbers of low-SR auditory fibers (in light green) decline during aging or following auditory damage, a significantly enhanced output of central circuits (central compensation and enhanced blue crosses) may critically depend on the maintained activity of high-SR auditory fibers (in orange), to assure the generation of high discharge rates and central compensation of deprived auditory input (ABR wave in blue). During this process of central neural gain (blue crosses), a BDNF- and memory–dependent amplification process requires the activation of hippocampal circuits (upper blue cross), the activation of the basal forebrain (BasF), the balancing activation of dorsolateral, medial prefrontal cortex (dlPFC and mPFC) and specific PFC regions, such as the inferior frontal gyrus (IFG), to enhance auditory signals above noise levels (feedback mechanism, blue downward arrow and cross on the right side). Modified after Knipper et al. (2020). IHC, inner hair cell; SGN, spiral ganglion neuron; SFR, spontaneous firing rate; HC, hippocampus; IFG, inferior frontal gyrus; BasF, basal Forebrain; PFC, prefrontal cortex; dlPFC, dorsolateral PFC; mPFC, medial PFC; AC, auditory cortex; PV, parvalbumin.

Numerous studies have indicated that acoustic trauma and age-dependent hearing loss are linked to reduced inhibition and enhanced excitation in ascending auditory circuits (Gerken, 1996; Milbrandt et al., 2000; Caspary et al., 2008; Wang et al., 2012; Ouda et al., 2015; Recanzone, 2018). Since low-SR auditory nerve fibers are vulnerable to noise damage and aging (Figure 3, low-SR in light green) (Heinz and Young, 2004; Heinz et al., 2005; Ruel et al., 2008; Kujawa and Liberman, 2009; Wang et al., 2012; Furman et al., 2013; Sergeyenko et al., 2013; Plack et al., 2014; Wu et al., 2019), deficits in this auditory nerve fiber type have been linked with temporal auditory discrimination deficits that follow acoustic trauma and age-related hearing loss in animals (Kujawa and Liberman, 2009; Plack et al., 2014; Wu et al., 2019) and humans (Liberman and Kujawa, 2017; Wu et al., 2019). Temporal auditory discimination deficits include those in spike timing and the synchronization of neural auditory responses that were shown to be required for following amplitude-modulated stimuli (Kuwada et al., 2002; Johnson et al., 2021). Auditory steady state responses are also an indicator for the proper processing of amplitude-modulated acoustic stimuli in subcortical areas and in the frontocentral cortex (Engelien et al., 2000). Previous studies indicated that during aging or after acoustic trauma, auditory response latencies can be shortened, and temporal coding, as measured through auditory steady state responses, enhanced (Möhrle et al., 2016; Eckert et al., 2021) when ABR wave IV is disproportionally elevated in response to a reduced ABR wave I (Figure 3, ABR wave in blue), a feature suggested to be linked to increased central neural gain (Box 2) (Figure 3, enhanced blue crosses).

In addition to low-SR auditory fiber processing sounds (Bharadwaj et al., 2015; Liberman, 2017), sustained fast (high-SR) auditory processing, is thus also critical for central neural gain and temporal auditory coding (Möhrle et al., 2017; Eckert et al., 2021). In line with this, computational models suggested that in response to deprived auditory input, the generation of sufficiently high discharge rates for centrally compensating homeostatic network changes may only work under conditions of preserved high-SR auditory nerve fibers (Schaette and Kempter, 2009). Diminished auditory input after acoustic trauma (Figure 3, crossed low-SR fibers contacting IHCs) has long been reported to possibly lead to a homeostatic network change and to an upregulation of neuronal responsiveness in central circuits (Salvi et al., 2000) (Figure 3, enhanced blue crosses). This homeostatic network change can be accompanied by a disproportional elevation of discharge rates as seen in the amplitude ratio of late ABR wave IV to early ABR wave I (Figure 3, ABR wave). Enhanced output relative to input in auditory neurons after acoustic trauma is suggested to be the result of disinhibition of neurons in the ventral or dorsal cochlear nucleus (Brigande and Heller, 2009; Cai S. et al., 2009; Schaette and Kempter, 2009; Schaette and McAlpine, 2011; Gu et al., 2012) or neurons of the IC (Gu et al., 2012; Heeringa and van Dijk, 2014). The subsequent hyperexcitability (Figure 3, high-SR in orange) spreads to the auditory cortex (Lu et al., 2011). The increased output of, e.g., CN neurons has been linked to steeper rate-level functions and a smaller dynamic range (Cai R. et al., 2009). As described for improved auditory perception, the process of accentuation of auditory stimuli that leads to central neural gain may require the co-activation of the basal forebrain to amplify stimulus-induced responses at subcortical and cortical levels (Figure 3, BasF blue downward arrow and cross) (Kilgard et al., 2002; Bajo et al., 2014; Kraus and White-Schwoch, 2015; Irvine, 2018a). Also, the activation of the inferior frontal gyrus (IFG), as part of the prefrontal cortex (PFC) (Figure 3, IFG), is crucial to retaining temporal and spatial associations of auditory events during auditory perception (Schonwiesner et al., 2007; Malmierca et al., 2014; Jafarpour et al., 2019). In general, the activation of PFC brain regions during perception is crucial to memorizing behaviorally relevant signals and increasing synaptic strength (Kraus and White-Schwoch, 2015; Weinberger, 2015; Irvine, 2018b). Particular and distinct medial (mPFC) and dorsolateral PFC (dlPFC) regions display crucial functions for basal inhibition of the hypothalamic-pituitary-adrenal (HPA) axis reactivity during central adjustment processes [review in Sullivan and Gratton (2002); Meltser and Canlon (2011), Canlon et al. (2013); de Kloet et al. (2014)de Kloet et al. (2019); Irvine (2018a), and Viho et al. (2019)]. Finally, in the auditory cortex, central neural gain control has been linked to feedforward inhibition, driven by the PV-IN, that spreads from the thalamus to the auditory cortex, eliciting amplified sound responses (Rabinowitz et al., 2012; Ji et al., 2016; Lohse et al., 2020; Pennington and David, 2020). The crucial role of PV-IN activation for central neural gain is emphasized through PV-IN potentiating drugs, which in the auditory cortex can trigger an enhanced signal-to-noise ratio (Deng et al., 2020). Optogenetic activation of PV-neurons, moreover, reduced spiking in the auditory cortex in general while enhancing functional connectivity (Hamilton et al., 2013). In the somatosensory and visual systems, an activation of PV-IN neurons is linked to enhanced stimulus-induced performance (Kim et al., 2016; Chen et al., 2017) that leads to enhanced response reliability, decreased signal variability, and improved reliability of signal information processing through an improved signal-to-noise ratio (Cardin et al., 2009; Sohal et al., 2009; Zhu et al., 2015) (Figure 3, Cortical resolution ⇑).

**FIGURE 4:**
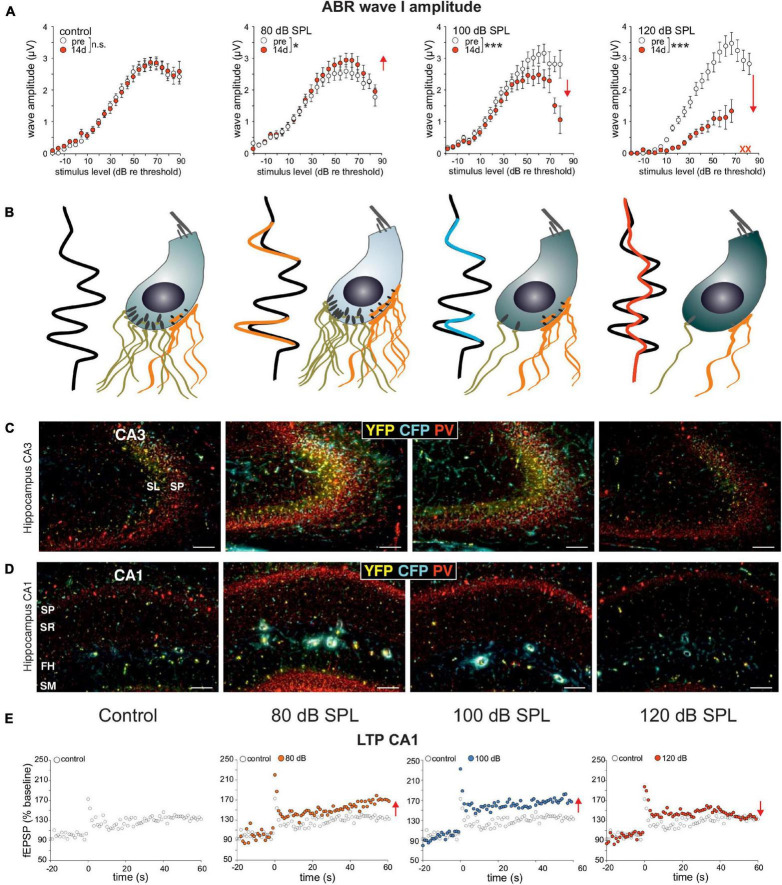
**(A)** ABR wave I responses are enhanced after sound exposure of 80 dB SPL and reduced after 100 dB SPL stimulation which can be compensated on the level of the ABR wave IV (see **B**), while ABR wave I amplitudes decrease after 120 dB SPL exposure. **(B)** ABR wave I amplitude changes are linked to changes in IHC ribbons that are mostly preserved after sound enrichment (80 dB SPL), but decline following mild acoustic trauma (100 dB SPL). In contrast, following severe stressful acoustic trauma (120 dB SPL), ribbon loss exceeds 50%, pointing to a loss of high-SR auditory fibers. **(C,D)** This goes along with marked increases in PV (red), *Bdnf* exon IV transcripts in capillaries (cyan), and exon VI transcripts in nerve endings (yellow), as can be observed in hippocampal CA3 **(C)** and CA1 **(D)** regions following 80 and 100 dB SPL, but not following 120 dB SPL exposure (Matt et al., 2018). **(E)** Significantly increased LTP observed after 80 dB SPL and 100 dB SPL, but not after 120 dB SPL sound exposure compared to that of the controls. Scale bars in **(B,C)** indicate 100 μm. Modified after Matt et al. (2018). SP, stratum pyramidale; SR, stratum radiatum; FH, fissura hippocampi; SM, stratum moleculare; SL, stratum lucidum.

Evidence that the activity-dependent BDNF recruitment may be part of this homeostatic central neural gain process (Figure 3, BDNF ⇑) came from experiments using BDNF-Live-Exon-Visualization (BLEV) reporter mice, generated to monitor the activity-dependent usage of BDNF from exon IV and exon VI. In these mice, stimulus-induced changes in *Bdnf* transcripts can be seen in nerve endings, glia cells, and capillaries (Matt et al., 2018; Singer et al., 2018b). This is in line with observations of activity-driven *Bdnf* transcripts shown for platelets (Chacon-Fernandez et al., 2016), capillary endothelial cells (Donovan et al., 2000), microglia, and astrocytes (Ferrini and De Koninck, 2013; Parkhurst et al., 2013). In BLEV mice 2 weeks after 80 dB SPL exposure, both wave I (Figure 4A) and wave IV (Matt et al., 2018) were elevated (=sustained elevation), whereas through mild acoustic trauma using 100 dB SPL exposure, wave I (Figure 4A) was reduced and wave IV (Matt et al., 2018) was unchanged (=centrally compensated) [see differences in Figures 4A,B, in control, 80 and 100 dB, (Matt et al., 2018)]. This was linked to elevated *Bdnf* exon IV/VI transcript levels both in the brainstem (Matt et al., 2018) and hippocampal CA3 region (Matt et al., 2018) (Figure 4C, yellow and cyan), associated with enhanced *Bdnf* exon IV transcripts in capillaries in the stratum lucidum (Figure 4C, cyan in SL). Also, PV-IN levels in perisomatic localization in the CA1 region were enhanced (Figure 4D, red) and linked with reduced PV-IN levels in dendritic localization [not shown, (Matt et al., 2018)], which together led to elevated hippocampal LTP (Figure 4E). When BLEV mice were exposed to stressful acoustic trauma of 120 dB SPL, however, that led to critically diminished numbers of high-SR auditory nerve fibers (judged from IHCs ribbon loss exceeding >50%), persistently reduced ABR wave IV amplitudes (Matt et al., 2018), failed recruitment of activity-dependent *Bdnf* transcripts, lowered hippocampal perisomatic PV-IN levels, and lower LTP levels were observed (Figures 4C–E, right panels) (Matt et al., 2018). This suggested that maintained fast (high-SR) auditory fiber processing is critical for central activity-dependent BDNF recruitment during homeostatic increased central neural gain. Particularly stressful acoustic trauma had, in previous studies, already been shown to lead to failed central neural gain that was linked to changes in hippocampal plasticity gene expression (Rüttiger et al., 2013; Singer et al., 2013; Matt et al., 2018).

Previous studies linked impaired *Bdnf* exon IV or VI transcripts with deficits in cognition and memory (Sakata et al., 2010; Vaghi et al., 2014; Mallei et al., 2015; Hill et al., 2016), together with deficits in cortical inhibition (Hong et al., 2008; Knipper et al., 2021), but this needs to be reconsidered in future studies with regard to deficiencies in the specific driving force for activating BDNF and inhibitory PV-IN activity.

## Altered Excitation and Inhibition in Acute Acoustic Trauma, Deafness, and Tinnitus: Lost Fast Auditory Processing

Hyperexcitability linked to reduced inhibition has also been observed in acquired deafness, congenital deafness, and tinnitus. The imbalance in excitation and inhibition in these auditory impairments is often interpreted as a compensatory response to auditory deprivation linked to increased central neural gain or an adaptive rewiring process. Here, we reconsider the imbalances of excitation/inhibition in these cases in the context of a loss of tonic inhibitory strength (Box 3), which can contribute to hearing disorders through decreased discharge population synchrony (enhanced variability) and a diminished signal-to-noise ratio following less developed or reduced fast (high-SR) auditory nerve fiber processing.

### Lost Fast Auditory Processing Following Acquired Deafness, Acoustic Trauma, or Tinnitus

Imbalances in excitation and inhibition are observed in acquired deafness, which can be caused by cochlear damage, middle-ear ossicle removal, acoustic trauma, or drug-induced deafness (Kotak et al., 2013; Mowery et al., 2019). In previous studies, it was shown that acquired deafness in mature animals led to hyperexcitability that coincided with a decrease in GABA and glutamic acid decarboxylase (GAD65) (Bledsoe et al., 1995; Abbott et al., 1999). Acquired deafness was linked with enhanced glutamatergic transmission, as shown in the superior olivocochlear complex or the midbrain (Potashner et al., 1997), with reduced glycinergic inhibition (Suneja et al., 1998; Potashner et al., 2000) or with decreases in GAD65 (Milbrandt et al., 2000). For the adult gerbil IC, it was shown that after monaural deafening, increased excitation occurred very quickly (McAlpine et al., 1997), even within a few minutes of deafening of the contralateral ear (Mossop et al., 2000). This fast time scale argues against a rewiring process or compensating refinement as causes of the enhanced excitability (Mossop et al., 2000). The rapid occurrence of increased excitation after deafening (Mossop et al., 2000) pointed rather to faster events, such as an acute switch of the GABAergic responsiveness from inhibitory to depolarizing activity (see also Section “Maturation of GABA-Responsive Neurons Prior to Hearing Onset”). Not yet analyzed in the short-term, a re-emergence of depolarizing GABAergic signaling and decline of KCC2 has been observed 3–30 days after auditory nerve transection (Tighilet et al., 2016). In addition, a rapid decline of KCC2 and a re-emergence of depolarizing GABAergic signaling has been observed within minutes during pathological epileptic firing (Khirug et al., 2010; Lee et al., 2011; Nardou et al., 2011), a feature that may be noted in future studies in the context of sudden deafness. Previous studies reported that a majority of subjects with acquired, single-sided sudden deafness experienced tinnitus on the affected side (Lee et al., 2017). Also, in patients with normal maturation of the auditory pathway who experienced acquired sudden sensorineural hearing loss, tinnitus regularly occurs, with a prevalence of 60–90%, often on the deaf side (Van de Heyning et al., 2008; Chadha et al., 2009; Eggermont and Kral, 2016). Not surprising in this context, tinnitus-inducing acoustic trauma has been linked with hyper-excitability and disinhibition, as observed in the cochlear nucleus (Dehmel et al., 2012; Koehler and Shore, 2013; Auerbach et al., 2014; Gao et al., 2016), in the IC (Chen and Jastreboff, 1995; Bauer et al., 2008), in the medial geniculate body (MGB) (Kalappa et al., 2014), or in the auditory cortex (Norena and Farley, 2013; Eggermont and Tass, 2015). In the majority of tinnitus studies, the elevated spontaneous activity, or hyperexcitability and reduced inhibition, was discussed in the context of an increased central neural gain [see reviews: (Schaette and Kempter, 2006, 2012; Norena, 2011; Schaette and McAlpine, 2011; Auerbach et al., 2014; Sedley et al., 2016; Shore et al., 2016; Roberts, 2018; Roberts and Salvi, 2019].

**FIGURE 5:**
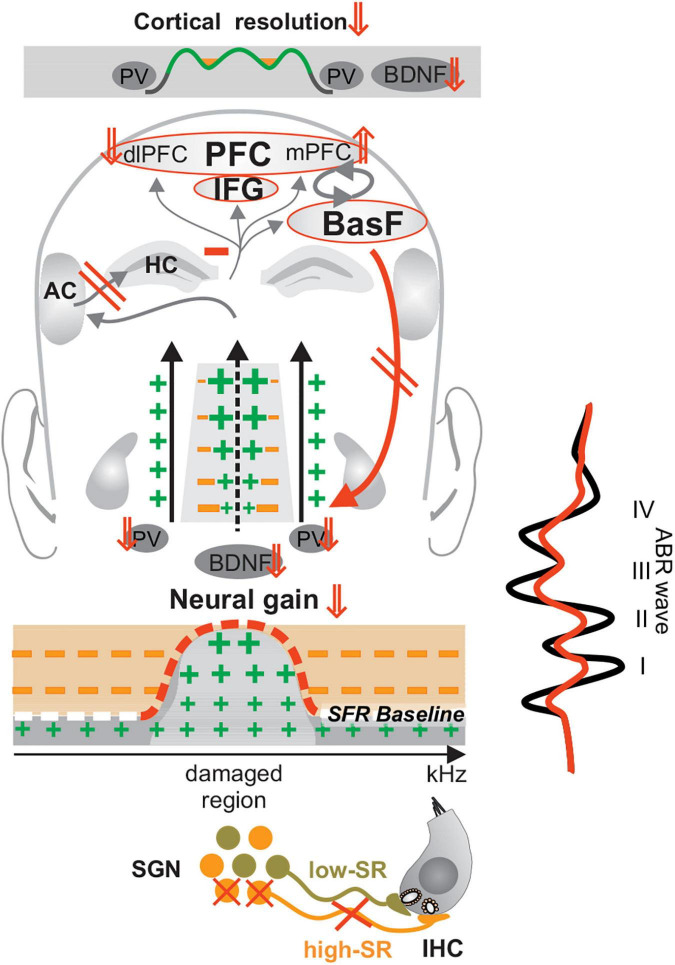
Lost fast auditory processing following acquired deafness, trauma or tinnitus. A critical loss of high-SR fiber (orange fibers) firing may promote the re-emergence of hyperexcitability (enhanced green crosses) in affected frequency regions through the loss of tonic inhibitory PV-IN activity (reduced orange minus) subsequent to a decrease of recruitment of activity-dependent BDNF. The subsequent elevation of basal spontaneous firing rates suggests an unbalanced prefrontal stress control (mPFC⇑, dlPFC⇓, negative feedback mechanism, and red downward arrow), which may contribute to a lack of compensation of altered auditory input (red ABR wave), further alertness and distress to, e.g., manifestations of phantom noise. Modified after Knipper et al. (2020). IHC, inner hair cell; SGN, spiral ganglion neuron; SFR, spontaneous firing rate; HC, hippocampus; IFG, inferior frontal gyrus; BasF, basal Forebrain; PFC, prefrontal cortex; dlPFC, dorsolateral PFC; mPFC, medial PFC; AC, auditory cortex; PV, parvalbumin.

Other studies showed that tinnitus is more linked to impaired homeostatic adjustment processes (Zeng, 2013; Knipper et al., 2015; Auerbach et al., 2019; Möhrle et al., 2019; Sedley, 2019) than to an increase in central neural gain [see for a review (Knipper et al., 2013, 2020, 2021; Zeng, 2020)]. This was first observed in rodent models of tinnitus (Rüttiger et al., 2013; Singer et al., 2013) and confirmed in patients (Hofmeier et al., 2018, 2021; Möhrle et al., 2019; Refat et al., 2021). In tinnitus patients, the delayed and reduced ABR wave V was shown to be accompanied by reduced blood-oxygen-level-dependent (BOLD) fMRI (functional Magnet Resonance Imaging) responses in the MGB, and in the primary auditory cortex and hippocampal regions (Hofmeier et al., 2018, 2021). It was speculated that a loss of fast auditory processing in the tinnitus frequency channels (Figure 5, crossed high-SR in orange) contributes through diminished tonic inhibitory strength (Box 3) of PV-IN (Figure 5, enhanced green crosses, reduced orange minus) to elevated response variability, reduced spike reliability and reduced signal-to-noise ratio (Zeng, 2020) in affected frequency regions (Figure 5, elevated SRF baseline red dashed line, enhanced green crosses). The relation of lost tonic inhibitory strength to reduced signal-to-noise ratio was, meanwhile, confirmed in numerous studies. Thus, e.g., optogenetic suppression of PV-IN activity, led to reduced task performance and reduced signal-to-noise ratio linked to increased baseline spontaneous gamma power and occlusion of changes in evoked gamma power (Chen et al., 2017). Also, a pharmacological PV-IN activation was previously shown to have the potential to diminish noise-induced tinnitus in animal studies (Deng et al., 2020). Moreover, reduced PV- density, but not somatostatin-positive interneurons density, in the primary auditory cortex was reported in tinnitus-perceiving animals (Masri et al., 2021). Regarding the tight correlation of BOLD to high-frequency gamma oscillations (Zumer et al., 2010; Butler et al., 2017), we thus speculate that the reduced and delayed ABR wave V and reduced BOLD fMRI responses in the auditory cortex observed in tinnitus patients (Hofmeier et al., 2018, 2021) may be the result of diminished tonic PV-IN strength, which through diminished discharge population synchrony, reduced spike reliability and enhanced spike variability (Cardin et al., 2009; Pi et al., 2013; Kim et al., 2016; Chen et al., 2017) may have contributed to an enhanced perception of internal noise (Knipper et al., 2020; Zeng, 2020).

If we question how, in the case of tinnitus, the internal noise can be heard as a disturbing sound, the observation becomes crucial that the tinnitus group exhibited not only reduced evoked fBOLD in the auditory cortex, but also elevated positive resting state connectivity (r-fcMRI) of default mode network activity, including the prefrontal cortex regions (PFC) (Hofmeier et al., 2018). In the tinnitus group, elevated r-fcMRI correlations were observed in the medial PFC (Hofmeier et al., 2018), a brain region said to be linked to stress excitation (McKlveen et al., 2013, 2016; Utevsky and Platt, 2014). This elevated r-fcMRI connectivity in mPFC correlated with the reduced sound-induced BOLD fMRI activity in the MGB (Hofmeier et al., 2018) and this reduced activity, in turn, correlated with increased latencies of the ABR wave V responses (Hofmeier et al., 2018) (Figure 5, mPFC⇑). Together, this points to an unbalanced extra-hypothalamic prefrontal (PFC) and hippocampal stress control (Sullivan and Gratton, 2002; Meltser and Canlon, 2011; Canlon et al., 2013; de Kloet, 2014; Irvine, 2018b; Viho et al., 2019). This unbalanced HPA stress control is suggested to contribute to further alertness and distress due to the phantom noise (Knipper et al., 2020, 2021).

Interesting in this context is that lower BDNF activation was previously associated with enhanced distress levels in tinnitus patients that suffered from BDNF Val^66^Met polymorphism (Vanneste et al., 2018). Also, reduced activity-dependent BDNF recruitment, linked with impaired *glucocorticoid receptor* phosphorylation was shown to lead to impaired long-term memory retention and to deficits in forming postsynaptic dendritic spines, for example after motor-skill training (Arango-Lievano et al., 2019). This means that diminished fast auditory processing (Figure 5, crossed high-SR fibers in orange) in distinct affected frequency regions could, through reduced activity-dependent BDNF (Figure 5, BDNF ⇓), lead to diminished PV-IN inhibitory strength (Figure 4, PV ⇓) and subsequent elevated SFR (Figure 5, SFR ⇑, red dashed line). The reduced activity-dependent BDNF recruitment in frontal brain regions would further diminish hippocampal responsiveness and diminish extra-hypothalamic prefrontal (PFC)/hippocampal stress control, and thus enhance alertness to the ‘brain noise.’ A previously suggested negative feedback of stress-receptor activation particular to fast auditory nerve response vulnerability (Singer et al., 2013, 2018a) (Figure 4) would accelerate the self-reinforcing downward spiral towards the increased stress and anxiety of tinnitus patients. Distress is, meanwhile, the best predictor of tinnitus severity, and a stronger predictor for tinnitus than any demographic factors (Crönlein et al., 2016; Beukes et al., 2021).

### Failed Maturation of Fast Auditory Processing Following Congenital Deafness

**FIGURE 6:**
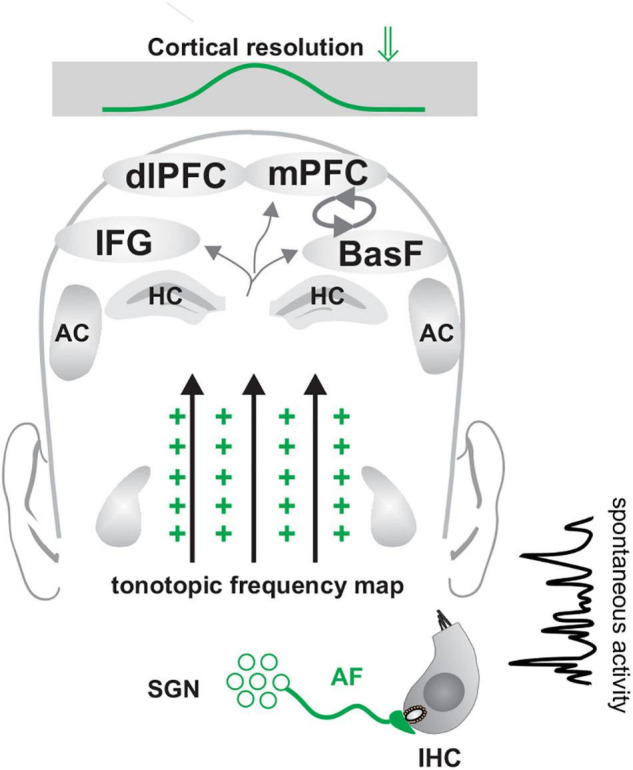
Enhanced excitability in the ascending auditory pathway (green crosses) in congenital deafness may reflect the maturational stage of the initial hyperexcitability (green crosses) in the ascending auditory pathway, when spontaneous firing dominates and fast (high-SR) auditory processing has not yet matured. During that time period, GABA-responsive neurons respond with depolarizing instead of hyperpolarizing activity. Modified after Knipper et al. (2020). IHC, inner hair cell; SGN, spiral ganglion neuron; HC, hippocampus; IFG, inferior frontal gyrus; BasF, basal Forebrain; PFC, prefrontal cortex; dlPFC, dorsolateral PFC; mPFC, medial PFC; AC, auditory cortex.

Numerous studies have analyzed hearing loss prior to hearing onset induced by kainate injection or ossicle destruction. Inhibitory neuronal markers were significantly diminished (Milbrandt et al., 2000; Mossop et al., 2000), whereas the excitability of various ascending central auditory neurons was significantly increased (Nordeen et al., 1983; Kitzes, 1984; Kitzes and Semple, 1985; Popelar et al., 1994). Also, cochlear ablation prior to hearing onset (Sanes et al., 1992) or deafness in the *deafness* (*dn/dn*) mutant mouse (Oleskevich and Walmsley, 2002) led to larger EPSP amplitudes and lower inhibitory synaptic strength. This phenomenon was observed in the cochlear nucleus (Oleskevich and Walmsley, 2002), in the lateral lemniscus, and in IC neurons (Sanes et al., 1992), as well as in thalamocortical and intracortical primary auditory cortex neurons (Kotak et al., 2005, 2013; Mowery et al., 2019). It was suggested that the larger EPSP amplitudes in congenital deafness may result from an increase in AMPA- and non-NMDA receptors and a decrease in inhibitory postsynaptic potential conductance. In another deaf *Vglut3*^–/–^ animal model, where glutamate release from IHCs is abolished due to deletion of vesicular glutamate transporter 3 (VGlut3) (Seal et al., 2008), elevated spontaneous activity, with longer bursts and smaller spikes, was recorded from cochlear (Babola et al., 2018; Sun et al., 2018) and from IC neurons (Babola et al., 2018). Since an enhanced excitability was seen in the IC neurons of VGlut3^–/–^ mice, even when the auditory cortex neurons were ablated, a top-down modulatory effect as the source for the enhanced central excitability could be excluded (Babola et al., 2018). In general, in these different cases of congenital deafness, the enhanced excitability in the ascending pathway were interpreted as an adaptive response to auditory deprivation (Babola et al., 2018) as a result of central rewiring (Nordeen et al., 1983; Moore, 1994), or as a compensatory response to the absence of synaptic activity (Davis and Bezprozvanny, 2001; Oleskevich and Walmsley, 2002) due to maladaptive central synaptic refinement (Ortmann et al., 2011). We propose that the enhanced excitability in the ascending auditory pathway in congenital deafness is neither the result of a long-term wiring process nor a compensatory response to the absence of central synaptic refinement, but rather may reflect inappropriate inhibitory shaping of auditory nerve fibers through efferent feedback control, possibly contributing to a failed switching of GABA-responsive neurons from depolarizing to hyperpolarizing activity prior to the onset of hearing (Lohrke et al., 2005) (Figure 6, see also Section “Maturation of GABA-Responsive Neurons Prior to Hearing Onset”). It may also indicate a failure of a proper maturation of fast (high-SR) auditory processing in the absence of auditory experience.

For congenital deafness in humans, this would inspire the question about a critical time period for the restoration of hearing through cochlear implants (CI); i.e., if not restored early enough, do the relevant auditory brain circuits remain in a stage of insufficient inhibitory strengths that hampers precise sharpening of receptive fields and proper inhibitory strength in the fine-grained microcircuits required for speech discrimination and temporal coding (Oxenham, 2018; Kral et al., 2019; Thompson et al., 2021)? The immediate onset of tinnitus that occurred in 60–90% of cases in children with cochlear implants when the implants were not in use (Van de Heyning et al., 2008; Chadha et al., 2009), may indicate that constant electrical stimulation through CIs is required to suppress ‘internal noise’ and to ‘silence’ phantom noise (Knipper et al., 2020). Recalling, moreover, that CIs in children are implanted on average at the age of 1–2 years (Peterson and Bergeson, 2015; Easwar et al., 2017), postponing the first auditory experience in these CI-carriers by 1–1.5 years (Sharma et al., 2002; Petersen et al., 2015) might induce a delay that is too long for some maturation steps. A judgment about a critical delay of auditory experience for proper implementation of distinct developmental steps may be assessed by looking at the prevailing deficits described in congenitally deaf CI-carriers. Deficits in CI-carriers include a reduction in binaural sound localization (Hamalainen et al., 2011; Lazard et al., 2012; Petersen et al., 2013; Slugocki and Trainor, 2014), missing left-hemisphere dominance (Petersen et al., 2013; Peterson and Bergeson, 2015; Easwar et al., 2017), weaker pitch sensitivity (Houtsma and Smurzynski, 1990; Kaernbach and Bering, 2001), lower dynamic range and higher thresholds (Sharma et al., 2002; Deroche et al., 2014), as well as lower mismatch negativity amplitudes, and prolonged CI-evoked cortical auditory evoked potential latencies (Ponton and Eggermont, 2001; Sharma et al., 2002).

To date, the latency of the auditory cortical component P_1_, which is used as an objective measure of developmental hearing experience (Sharma et al., 2005a,b), were reported to be shorter in early-implanted deaf children as compared to late-implanted children (Sharma et al., 2005a). This already points to a critical time window of CI implantation to achieve temporal precise hearing. The less variable performance, the reduced expansion of activated areas at the primary auditory cortex, and less exuberant connections between the visual cortex and auditory cortex in early- versus late-implanted congenitally deaf cats (Lomber et al., 2010; Land et al., 2016; Kral et al., 2019) point to critical time windows for CI implantation. In such cases, a possibly immature stage of cortical inhibitory shaping with incompletely accomplished clustering and pattern segregation of auditory-specific modalities may be considered.

It is likely that documented deficits in CI-carriers, such as in latency shift, sound localization, or pitch sensitivity, may critically depend on fast auditory processing and possibly on proper high-SR auditory fiber processing. Even missing left hemisphere dominance (Petersen et al., 2013; Peterson and Bergeson, 2015; Easwar et al., 2017) may be related to the strong impact that neuronal activity and sensory experience is predicted to have on the proliferation and differentiation of oligodendrocytes during myelination (Xin and Chan, 2020). Keeping this in mind, there is a distinct need for the influence of fast (high-SR) auditory processing on myelination progress to be urgently tested in future studies. In the course of hearing restoration through successful implementation of CIs or hearing aids, attempts should be made to monitor the implementation of proper inhibitory strength.

## Altered Excitation and Inhibition Following Diminished Fast Auditory Processing Linked to ‘Central’ Hearing Loss

### Failed Fast Auditory Processing in Autism Spectrum Disorders

An excitation/inhibition imbalance is also considered to be a characteristic feature of ASD, which is accompanied by reduced PV-IN labeling (Takano and Matsui, 2015; Pirone et al., 2018; Goel et al., 2019), elevated levels of the activity-related gene Arg3.1/Arc (Korb and Finkbeiner, 2011; Goel et al., 2019; Eckert et al., 2021), or by increased fEPSPs (Mohn et al., 2014). A reduced inhibition linked with reduced levels of GABA-synthetisising enzymes and GABA receptors was observed in the brain of patients with ASD (Fatemi et al., 2002, 2009, 2010; Reynell and Harris, 2013; Schur et al., 2016; Cukier et al., 2020). In autism patients and animal models, the reduced inhibition is said to reduce reliability (increasing variability) of signal transformation and the signal-to-noise ratio (Dinstein et al., 2012; Haigh et al., 2016). Interestingly, deficits in fast auditory processing are also reported in nearly normal-hearing children that have ASD (Fitch et al., 2013; Foss-Feig et al., 2017), and here, deficits in fast auditory processing are linked to markedly delayed and displaced auditory steady-state responses (Stroganova et al., 2020), or with rapid spectral-ripple discrimination deficits (Ankmnal Veeranna et al., 2019).

A previous study in a mouse model with a cell-specific deletion of *Bdnf* in Pax2 positive GABAergic precursor cells (*Bdnf^Pax2^*KOs mice) showed an autism-like phenotype (Eckert et al., 2021). These mice exhibited normal basal hearing function, but with reduced and delayed ABR wave IV, diminished PV-IN labeling in the auditory cortex and hippocampus, and with reduced tonic inhibitory strength and elevated spontaneous firing rates in dorsal cochlear nucleus (Eckert et al., 2021) and IC neurons (Chumak et al., 2016). These features were associated with a reduced (sound)-induced LTP/LTD adjustment, impaired learning, deficits in social behavior, and enhanced anxiety and stress levels (Eckert et al., 2021). This phenotype thus pointed to a diminished extra-hypothalamic stress control (de Kloet et al., 2019). Impaired PV-IN mediated inhibitory shaping of auditory and hippocampal circuits, as observed in *Bdnf^Pax2^*KOs, was moreover suggested to lead to impaired central neural gain after sound enrichment, deficits in LTD, and pathologically increased activity-related gene Arc expression (Eckert et al., 2021). Proper LTD and balanced Arc expression levels are crucial for the control of fast changes in AMPA receptor trafficking during novelty discrimination (Derkach et al., 2007; Waung et al., 2008; Blair et al., 2019; Penrod et al., 2019; Roth et al., 2020).

This finding emphasizes that deficits in fast auditory processing, leading to diminished tonic inhibitory strength, can impair central neural gain and affect not only temporal coding but also cognitive functions, including novelty discrimination tasks and learning.

Deficits in fast auditory processing may be uniquely critical in the auditory system, which in comparison to other senses relies particularly on narrow time windows and a high speed of information flow (Zajac and Nettelbeck, 2018). To further validate a causal relationship between failed maturation of fast auditory processing and the autism phenotype, it will be necessary to explore in more detail the fine-structure of ABR and auditory steady-state responses, in combination with functional electroencephalography (EEG), and fMRI in animal models and children with autism-spectrum disorders.

### Failed Fast Auditory Processing During Age-Dependent ‘Central’ Hearing Loss

**FIGURE 7:**
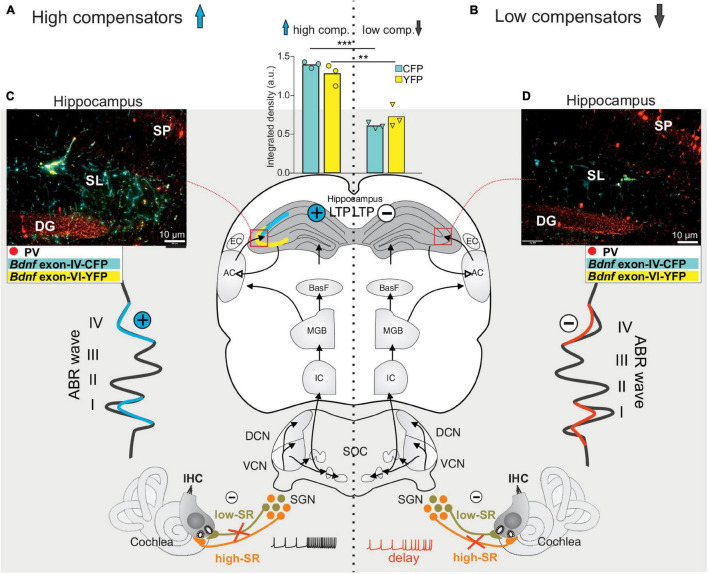
Schematic presentation of high and low central compensatory mechanisms in the aging auditory system. **(A)** Independent of aging or differences in hearing thresholds, cochlear synaptopathy can differ, depending on whether de-afferentation due to low-SR auditory nerve fiber loss dominates (**A**, low-SR in light green) or high-SR auditory nerve fiber loss dominates (**B**, high-SR in orange). In the first case, an ABR wave I reduction is associated with disproportionally increased ABR wave IV amplitude (**A**, left blue amplitude, plus in blue circle), with elevated *Bdnf* exon IV (cyan) and exon VI (yellow) expression in hippocampal circuits (**C**, left panel and bar graph) and higher hippocampal LTP (plus in blue circle). In the case of a critical loss of fast (high-SR) auditory nerve fiber processing (**B**, high-SR in orange), an attenuated temporal resolution capacity of auditory nerve fibers (**B**, delay) is associated with permanently decreased ABR wave IV amplitude (**B**, red amplitude, minus in white circle), decreased recruitment of hippocampal *Bdnf* exon IV and exon VI transcripts (**D**, right panel and bar graph) and significantly lower LTP mobilization (minus in white circle). ABR, auditory brainstem response; IHC, inner hair cell; VCN, ventral cochlear nucleus; DCN, dorsal cochlear nucleus; SOC, superior olivary complex; IC, inferior colliculus; MGB, medial geniculate body; BasF, basal Forebrain; AC, auditory cortex; EC, entorhinal cortex; PV, parvalbumin.

A link between deficits in fast auditory processing and age-related deficits in cognition has previously been proposed. Thus, studies analyzing aging animals showed that, independently of age or hearing thresholds, animals fell into two groups regarding central auditory responses to cochlear synaptopathy: The ‘high-compensating’ group was able to respond to cochlear synaptopathy with an enhanced input/output function (elevated ABR wave IV/I ratio), linked with enhanced LTP and maintained temporal processing (Eckert et al., 2021) (Figure 7A, left panel). The other, the ‘low-compensating’ group, exhibited weakened compensatory capacity (lower ABR wave IV/I ratio), linked with lower LTP, and weakened temporal coding (Marchetta et al., 2020) (Figure 7B, right panel). The reduced capacity to centrally compensate age-dependent cochlear synaptopathy, and the lower hippocampal LTP with attenuated temporal coding, was associated with a prolonged latency of the auditory nerve response (ABR wave I) in comparison to the high-compensating group (Marchetta et al., 2020), suggesting that fast (high-SR) auditory processing was mitigated in this group. In the ‘low-compensating group,’ moreover, lower levels of *Bdnf* IV and VI transcripts were seen in hippocampal nerve terminals and capillaries in comparison to the high-compensating group, (Figures 7C,D, compare yellow and cyan staining). Although differences in auditory response latencies, auditory neural responses to modulated tones, and LTP may point to differences in inhibitory strength following differential impairment of fast auditory fiber processing (Marchetta et al., 2020), experimental evidence for this is currently missing. Reduced GABAergic activity was, however, previously observed in ascending auditory circuits, e.g., during aging, a phenomenon that was hypothesized to be linked to cognitive decline (Ibrahim and Llano, 2019; Pal et al., 2019; Rogalla and Hildebrandt, 2020). In these cases also, it may be useful to consider deficits in fast auditory processing as being causally related to age-dependent hearing loss that is associated with cognitive deficits.

## Coupling of Inhibitory/Excitatory Circuit Activation to Cerebral Blood Flow

### The Role of GABAergic Activity for Neurovascular Coupling

In questioning whether reduced tonic inhibitory strength following fast auditory processing may be particularly critical for cognition, as predicted from autism animal models (see Section “Failed Fast Auditory Processing in Autism Spectrum Disorders”), the critical time period of maturation of fast auditory processing and inhibitory strength in auditory and associated limbic circuits - between the 2nd and 3rd postnatal week in rodents (Itami et al., 2007; Eckert et al., 2021) needs to be reconsidered. In rodents, this time period overlaps with the time of progressively faster BOLD signals, in which brain regions manifest an increased intensity in response to sensory stimulation (Colonnese et al., 2008). Thus, before P11 in rodents (prior to hearing onset), brain activation is not associated with sustained increases of the cerebral blood flow (CBF), which would result in none or a negative BOLD signal (Colonnese et al., 2008; Kozberg et al., 2013; Iadecola, 2017). Only in the 2nd and 3rd week does neural activity lead to increasingly faster and more intense hemodynamic responses, as shown by BOLD fMRI (Colonnese et al., 2008; Iadecola, 2017). The increased hemodynamic BOLD fMRI response during this critical time period is linked with pronounced neurovascular and systemic changes, including increases in vascular density, synaptogenesis, energy metabolism, and sensitivity of the cerebral microcirculation to vasoactive stimuli (Nehlig et al., 1989; Colonnese et al., 2008; Goyal et al., 2014; Engl et al., 2017; Iadecola, 2017). The time of increased hemodynamic BOLD fMRI responses is also the time when in rodents, long-term potentiation in the hippocampus gradually matures (Ostrovskaya et al., 2020).

We questioned whether the maturation of fast (high-SR) auditory processing, of inhibitory PV-IN microcircuits, of hemodynamic BOLD fMRI responses, and of LTP may not only be correlated in time, but also functionally. More precisely, we asked whether the imbalances in excitation and inhibition in hearing disorders that correlated with reduced tonic inhibitory strength, as predicted in the case of impaired fast auditory nerve fiber responses (Chumak et al., 2016; Eckert et al., 2021), might also have implications for hemodynamic responses.

This hypothesis is based on new insights into the mechanism of hemodynamic responses: Previously, glutamatergic neuronal activity was assumed to mainly trigger hemodynamic responses and vasodilation during a bilateral homeostatic response: Glutamatergic neuronal activity, such as neural feedforward signaling, includes neuronal-derived nitric oxide (NO) release from the glutamatergic synapses that causes a metabolic feedback signal in smooth muscle cells of parenchymal arterioles, finally leading to vasodilation [for a review see Attwell et al. (2010); Kisler et al. (2017); Ledo et al. (2021)]. Newer findings, however, suggest that arteriole vasodilation may possibly occur independently of NO (Chow et al., 2020). In line with this, neurovascular coupling is preserved in mice lacking endothelial NO synthase (Girouard et al., 2007). Also, a release of NO from GABAergic interneurons was shown to affect the hemodynamic responses through the nitric-oxide sensitive guanylyl cyclase (NOsGC) pathway (Cauli et al., 2004; Kocharyan et al., 2008; Lee et al., 2020). These observations were corroborated by experiments employing an optogenetic activation of GABAergic interneurons, which provoked a significant increase in the CBF (Uhlirova et al., 2016; Iadecola, 2017; Vazquez et al., 2018). Also, optogenetic activation of GABAergic interneurons increased CBF even when glutamatergic GABAergic activity was pharmacologically blocked (Anenberg et al., 2015). Fast PV-IN are decisive in generating gamma-oscillations, as measured with EEG in combination with optogenetic techniques to stimulate PV-INs (Cardin et al., 2009; Sohal et al., 2009; Chen et al., 2017). Thus previous studies that found a significantly reduced gamma activity following stress events that lead to impaired neurovascular coupling (Sohal et al., 2009; Lee et al., 2015; Chen et al., 2017; Han et al., 2019) would also support the involvement of PV-IN GABAergic signaling on CBF. Interestingly, in this case, reduced GABAergic activity after stress occurred in nNOS-positive interneurons (Czeh et al., 2015, 2018; Csabai et al., 2018; Han et al., 2019), underscoring PV-IN activity as possibly contributing to NO-induced vasodilation.

The contradicting assumptions, that on the one hand NO-release from GABA-IN may influence endothelia cells of blood vessels, and thereby change their diameter (Lee et al., 2020), while on the other hand arteriole vasodilation is suggested to occur independently of NO (Chow et al., 2020), may moreover find a rational solution through suggestions that put capillary dilation in the focus of hemodynamic responses, rather than smooth-muscle-cell arteriole dilation. Thus, capillary flow was recently suggested not to be a passive consequence of the flow in upstream smooth muscle-unsheathed arterioles, but vice versa; capillary dilation may be a primary event preceding arteriole dilatation (Kisler et al., 2017). In this scenario, capillary dilation would occur as a result of the relaxation of pericytes, and this local dilation would spread from capillaries toward larger arterioles in a secondary step (Hall et al., 2014; Lendahl et al., 2019; Han et al., 2020). A crucial role of pericytes for capillary vasodilation during hemodynamic responses has been shown in numerous previous studies (Lourenco et al., 2014; Sweeney-Reed et al., 2016; Caporarello et al., 2019; Alarcon-Martinez et al., 2020), although others failed to demonstrate this (Fernandez-Klett et al., 2010; Hill et al., 2015; Wei et al., 2016; Cudmore et al., 2017; Iadecola, 2017). On the other hand, pericytes have been shown to express NO-responsive enzymes (Friebe et al., 2018). Also, a pericyte-induced role for vasodilation through capillaries would become feasible, since the large surface area of capillaries and minimal changes in their diameter would produce a large change in blood flow (Han et al., 2020).

It is thus challenging to consider that the increase in PV-IN mediated inhibitory strength between the 2nd and 3rd postnatal week (Itami et al., 2007; Eckert et al., 2021) and the coinciding progressive changes in shape and intensity of BOLD signals (Colonnese et al., 2008) are functionally related events. While all these findings may support the notion that PV-IN have a potential to modulate CBF, evidence for their participation in neurovascular coupling to physiological stimuli is still limited. We may, however, conclude that, in addition to glutamatergic neuronal activity influences on vasodilation (Attwell et al., 2010; Kisler et al., 2017; Ledo et al., 2021), PV-IN GABAergic activity may play a role in hemodynamic responses.

## Conclusion

**FIGURE 8:**
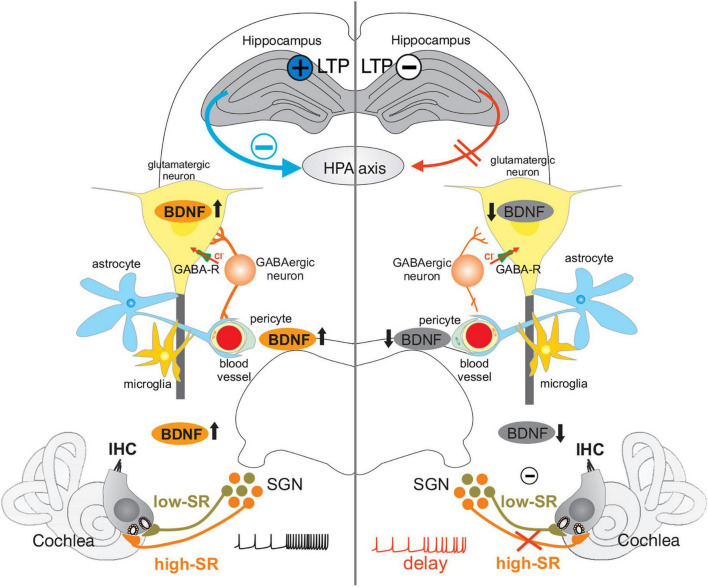
Under healthy conditions **(Left)**, fast auditory processing (high-SR in orange) enables context-specific information processing in auditory and associated circuits (hippocampus, HPA axis) through upregulation of activity-dependent BDNF (↑). Increased central neural gain, represented by increased hippocampal LTP (black cross in blue circle), allows the facilitation of BDNF/PV-IN circuits by BDNF ↑ in glutamatergic neurons, and an increase in perisomatic inhibitory strength (GABAergic neuron contacting glutamatergic neuron) dependent on increased stimulus responses. These in turn provide a balanced HPA axis control (blue arrow and minus) and local hemodynamic supply (blood vessels) for a long-lasting improved signal-to-noise ratio and stimulus discrimination above noise (black spike train). During aging, or following acoustic trauma, acute, or congenital deafness **(Right)**, a critical loss of high-SR auditory nerve fibers (orange) occurs, leading to reduced auditory driving force and BDNF expression (↓). This is associated with reduced hippocampal LTP (black minus in white circle), reduced tonic-PV-IN strength (reduced synaptic contacts of GABAergic neurons on glutamatergic neurons), altered extra-hypothalamic stress control (red arrow to HPA axis), and possibly disturbed coupling of inhibitory neuronal activity to hemodynamic responses, leading to altered sound processing (red spike train). IHC, inner hair cell; SGN, spiral ganglion neuron; GABA-R, GABA receptor; HPA axis, hypothalamic-pituitary-adrenal axis; LTP, long-term potentiation; BDNF, brain-derived neurotrophic factor.

During aging or following acoustic trauma, acute or congenital deafness, a critical diminution of fast (high-SR) auditory driving force diminishes activity-dependent BDNF activities and tonic-PV-IN strength, hippocampal LTP, extra-hypothalamic stress control and possibly proper coupling of inhibitory neuronal activity to hemodynamic responses, accelerating a negative feedback cycle (Figure 8, right side). Under healthy conditions (Figure 8, left side), when critical fast auditory processing is maintained, context-specific information through specific activation of BDNF signaling in auditory and associated circuits, allow, through increased central neural gain, the facilitation of BDNF/PV-IN dependent increase stimulus responses that in turn guarantee a balanced HPA axis control and local hemodynamic supply for a long-lasting improved signal–to-noise ratio and stimulus discrimination above noise.

In view of the increasing evidence of a link between hearing loss and dementia, a better understanding of this possible relationship is an important challenge (Livingston et al., 2017; Griffiths et al., 2020; Montero-Odasso et al., 2020). We suggest here that a differential role of auditory fiber processing for specific imbalances in excitation/inhibition can be regarded as a key signature of hearing disorders with or without cognitive decline.

## Author Contributions

MK designed, wrote, and revised the manuscript. WS wrote the manuscript and made the figures. KS, GEH, LR, CB, and RL wrote and revised the manuscript. YLH wrote the figure legends and helped writing the manuscript. All authors contributed to the article and approved the submitted version.

## Conflict of Interest

The authors declare that the research was conducted in the absence of any commercial or financial relationships that could be construed as a potential conflict of interest.

## Publisher’s Note

All claims expressed in this article are solely those of the authors and do not necessarily represent those of their affiliated organizations, or those of the publisher, the editors and the reviewers. Any product that may be evaluated in this article, or claim that may be made by its manufacturer, is not guaranteed or endorsed by the publisher.
